# Association of oral health with various work problems: a cross-sectional study of Japanese workers

**DOI:** 10.1186/s12903-023-03196-4

**Published:** 2023-07-15

**Authors:** Satomi Shimada, Takashi Zaitsu, Akiko Oshiro, Shiho Kino, Jun Aida

**Affiliations:** Department of Oral Health Promotion, Graduate School of Medical and Dental Sciences, Tokyo Medical and Dental University, 1-5-45 Yushima, Bunkyo-Ku, Tokyo, 113-8510 Japan

**Keywords:** Oral health, Work problems, QOL, Epidemiology, Logistic regression, Presenteeism

## Abstract

**Background:**

Oral diseases affect quality of life and known to decrease productivity. We examined the impact of oral health status on various types of work problems.

**Methods:**

This cross-sectional study used data from an internet-based self-report questionnaire survey administered to workers in Japan. Responses to the questionnaire regarding seven types of oral health-related work problems (1. Stress; 2. Lack of focus; 3. Lack of sleep; 4. Lack of energy; 5. Lack of communication due to halitosis; 6. Lack of communication due to appearance; 7. Lack of ability due to dental-related pain) were investigated and statistically analyzed. Explanatory variables were self-reported oral health status, number of teeth, and gum bleeding. To examine the association of oral health with the presence of work problems, logistic regression analysis was used to estimate the odds ratio (OR) and 95% confidence interval (CI). Age, sex, educational attainment, income, the presence of diabetes, and industrial classifications were used as the covariates.

**Results:**

A total of 3,930 workers (mean age: 43.3 (SD = 11.7), 2,057 males and 1,873 females) were included. Overall, a total of 6.2% of workers reported having at least one oral health-related work problem in the past year, whereas 21.8% of those with poor self-reported oral health reported work problems. Workers with poor self-reported oral health were 3.58 (95% CI (1.70–7.56) times higher odds of reporting work problems than those with excellent self-reported oral health.

**Conclusions:**

Oral health was found to be associated with various work problems. Oral health promotion policies are needed in the workplace.

**Supplementary Information:**

The online version contains supplementary material available at 10.1186/s12903-023-03196-4.

## Background

Oral health lead to serious health problems and have a negative impact on oral health-related quality of life. Oral health is known to be related to health conditions and stress, which may affect work performance. Dental pain has a considerable impact on quality of life and can lead to poor work performance [1, 2]. Poor dental appearance and halitosis are related to psychological stress and depression and can make individuals reluctant to engage in social communication in the workplace, thus adversely affecting their work performance [3, 4]. Significant oral symptoms and problems also adversely affect dietary lifestyle and nutrition intake [5]. Individuals with oral problems may experience a decline in their oral health related quality of life [6, 7].

Previous studies have reported that systemic health conditions and stress affect work performance [8–10]. Since oral health is known to be related to health conditions and stress [11–15], it also possibly affects work performance [16, 17]. However, the impact of oral health on work performance has rarely been addressed. The economic impact of dental diseases on society encompasses both direct and indirect costs. The direct costs are attributed to dental treatment by dental professionals, whereas the indirect costs are attributed to time loss from work and activities due to dental problems and treatment [18–20]. Early studies have demonstrated that $187.61 billion was lost worldwide in 2015 due to productivity loss, representing the time loss caused by the treatment of oral symptoms and diseases [21].

Recent critical measures of poor work performance encompass absenteeism and presenteeism; absenteeism is defined as an absence from work due to a disease or an accident, whereas presenteeism is defined as the physical presence of workers with dysfunctional conditions induced by health problems in the workplace [16]. In addition to work absenteeism, work presenteeism reduces work productivity [22]. Although the association between oral health status and work presenteeism has been previously reported [16, 17], few studies have examined the details of work participation, such as the type of productivity loss caused by oral health problems. As oral health has various functions, there is a possibility that poor oral health affects various types of work presenteeism. Determining the adverse effects of oral health problems on work performance will provide recommendations for reducing productivity loss due to oral health problems.

Therefore, the aim of this study was to examine the association of oral health status with various types of work problems. We hypothesized that oral health related work problems were prevalent among the workers with poor oral health status.

## Methods

### Study settings

Ethical approval for this study was obtained from the Ethics Review Committee of the School of Dentistry, Tokyo Medical and Dental University (Approval number D2015-526). This cross-sectional study used data from an internet-based, self-report questionnaire survey conducted in March 2017 among workers in Japan. Participants were originally recruited through a service managed by company M. Written informed consent was obtained at the time of registration. Data were collected from equal numbers of male and female workers from each of the 11 major classifications of the Ministry of Internal Affairs and Communications. Among 4000 workers, 3930 agreed to participate in the questionnaire survey.

### Outcome variables

Oral health-related work problems were used as the outcome variables. The survey inquired about the presence of work problems due to oral symptoms or diseases through the following question: “In the past year, have you had problems with your work due to oral diseases or symptoms?”. Participants were also asked to indicate the presence of specific work problems by responding to the following statements: 1) I felt stressed, which affected my work; 2) I could not concentrate at work; 3) I couldn’t sleep at night and it affected my work the next day; 4) I felt a lack of energy and vitality; 5) I couldn’t talk to others because I was worried about my bad breath; 6) I was so worried about the appearance of my teeth and mouth that I couldn’t stand to go out in public; and 7) The pain interfered with my ability to work. These seven work problems were defined as follows: 1) stress; 2) lack of focus; 3) lack of sleep; 4) lack of energy; 5) lack of communication due to halitosis; 6) lack of communication due to appearance; and 7) lack of ability to work due to dental-related pain. The responses to these questions were scored on a 5-point scale (from 1: a great impact to 5: no impact), and the responses were then dichotomized (no impact/any impact). The distribution of the 5-point scale was showed in Additional Table 1.

### Explanatory variables

Single-item self-reported oral health (SROH) status, number of teeth, and gum bleeding were used as the explanatory variables. SROH was asked, with the following questions; “What is your condition of your teeth and gums?” [23], and participants chose from one of the following options; “excellent”, “very good”, “good”, “fair”, and “poor”. The number of teeth was asked by “How many teeth do you have, including covered teeth (gold and silver), crowns, and remaining roots?”, and participants chose “19 or fewer teeth” or “20 or more teeth”. Previous studies have reported that having 20 or more teeth was considered to have sufficient masticatory function [24, 25]. The inquiry regarding gum bleeding was as follows: “Do you have bleeding gums while brushing your teeth?”. Participants chose one of the following options to indicate their response: “always”, “sometimes” and “never”.

### Covariates

We selected the covariates based on previous studies [26–28]. Sociodemographic information, the presence of diabetes, and industrial classifications were the covariates in this study. The sociodemographic information included age, sex, educational attainment (elementary and secondary school graduate, vocational school or junior college graduate, university graduate, master's degree or doctoral degree, other), and income (< 2 million yen, ≥ 2 to < 4 million yen, ≥ 4 to < 6 million yen, ≥ 6 to < 8 million yen, ≥ 8 million yen, unknown). According to the Japanese Standard Industrial Classification 12^th^ revision [29], the occupations of the participants were categorized into 20 types: (1) agriculture and forestry; (2) fisheries; (3) mining and quarrying of stone and gravel; (4) construction; (5) manufacturing; (6) electricity, gas, heat supply and water; (7) information and communications; (8) transport and postal services; (9) wholesale and retail trade; (10) finance and insurance; (11) real estate and goods rental and leasing; (12) scientific research, professional and technical services; (13) accommodation and eating and drinking services; (14) living-related and personal services and amusement services; (15) education, learning support; (16) medical, health care and welfare; (17) compound services; (18) services (not otherwise classified elsewhere); (19) public duties (excluding those classified elsewhere); and (20) industries that unable to classified. Of these, we defined (1) to (2) as “primary industries”, (3) to (5) as “secondary industries”, and (6) to (20) as “tertiary industries” [26].

### Statistical analysis

**Fig. 1:**
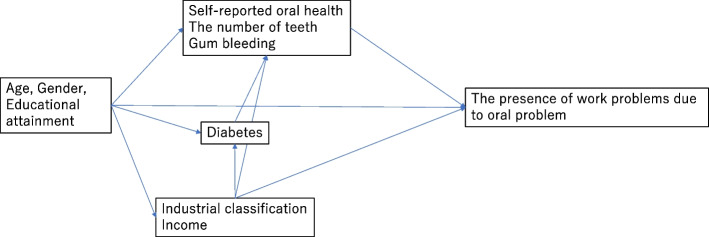
Directed acyclic graph for logistic regression analyses of the present study

Logistic regression analysis was used to examine the association of oral health with the presence of work problems, and to estimate the odds ratio (OR) and 95% confidence interval (CI). Figure 1 shows the directed acyclic graph (DAG) for the logistic regression analysis. We conducted univariable and multivariable analyses. First, as the outcome variable, the presence of work problems due to oral symptoms and diseases was used in the analysis. Then, the seven specific work problems were used as the outcome variables. In addition, to perform sensitivity analyses, we used a 5-point scale for seven specific work problems as the outcome variables rather than the dichotomized scale. Ordered logistic regression was applied to these sensitivity analyses (Additional Tables 2-4). All data analyses were performed using STATA MP ® 17.0 (Stata Corporation, College Station, Texas, USA). *P* < 0.05 was considered indicative of statistical significance.

## Results

Table 1 shows the descriptive distribution of oral conditions by age, sex, educational attainment, income, the presence of diabetes, and industrial classifications. The participants in this study included 3,930 (2,057 males and 1,873 females) workers. The mean age of the participants was 43.3 ± 11.7 years. Among the participants, 244 (6.2% of the total) reported that oral problems interfered with their work.

**Table 1:** Descriptive distribution of the oral conditions by age, gender, education, income, and systemic diseases (*n* = 3930)

	n (%)	Self-reported oral health (%)					Number of teeth (%)		Bleed when brushing teeth (%)		
	Total	Excellent	Very good	Good	Fair	Poor	19 or fewer teeth	20 or more teeth	Always	Sometimes	None
Age											
Under 30	625 (15.9)	23.5	17.9	14.7	14.8	7.0	9.4	17.0	20.8	18.2	13.9
30〜39	969 (24.7)	30.3	27.0	24.8	20.4	20.4	17.1	25.9	28.9	27.8	22.0
40〜49	1011 (25.7)	22.8	26.1	26.2	24.9	29.6	24.6	25.9	24.5	25.5	26.0
50〜59	888 (22.6)	14.7	19.7	22.8	27.1	29.6	29.5	21.4	18.2	19.2	25.4
Over 60	437 (11.1)	8.8	9.3	11.5	12.8	13.4	19.4	9.7	7.5	9.3	12.7
Sex											
Male	2057 (52.3)	45.3	50.3	53.4	54.6	54.9	62.5	50.7	56.0	51.8	52.4
Female	1873 (47.7)	54.7	49.7	46.6	45.4	45.1	37.5	49.3	44.0	48.2	47.6
Educational attainment											
1*	1416 (36.0)	30.3	27.2	37.1	42.9	53.5	45.9	34.4	52.2	36.8	34.2
2*	839 (21.3)	20.2	22.4	22.2	19.8	16.2	18.9	21.8	17.0	20.7	22.2
3*	1675 (42.6)	49.5	50.4	40.8	37.2	30.3	35.2	43.9	30.8	42.5	43.6
Income											
2 < million yen	577 (14.7)	11.7	12.8	15.0	16.9	16.9	15.1	14.6	18.9	15.6	13.7
2 ≥ to < 4 million yen	1479 (37.6)	37.1	35.5	38.2	38.7	39.4	39.1	37.4	39.0	37.9	37.3
4 ≥ to < 6 million yen	810 (20.6)	20.2	23.3	19.6	19.7	21.1	21.9	20.4	18.2	22.0	19.8
6 ≥ to < 8 million yen	299 (7.6)	9.8	9.7	6.0	7.6	7.7	8.9	7.4	8.8	6.7	8.2
8 ≥ million yen	210 (5.3)	4.6	6.3	5.7	4.6	1.4	4.1	5.6	1.9	3.9	6.7
Unknown	555 (14.1)	16.6	12.3	15.6	12.5	13.4	10.9	14.7	13.2	13.9	14.3
Diabetes											
Absence	3806 (96.8)	98.0	98.8	96.5	95.0	95.8	93.2	97.4	93.7	97.6	96.5
Presence	124 (3.2)	2.0	1.2	3.5	5.0	4.2	6.8	2.6	6.3	2.4	3.5
Industry											
Primary industry	305(7.8)	10.4	6.8	7.9	7.7	7.0	9.8	7.4	7.5	7.2	8.2
Secondary industry	1097(27.9)	24.8	26.6	28.5	28.8	31.7	29.7	27.6	33.3	27.8	27.6
Tertiary industry	2528(64.3)	64.8	66.7	63.6	63.5	61.3	60.5	65.0	59.1	65.0	64.2

1* Elementary and secondary school graduate

2* Vocational school or junior college graduate

3* University graduate, master's degree or doctoral degree, other

Table 2 shows the prevalence (%) of oral health related work problems by oral status (*n* = 3930). On average, 6.2% of the workers had experienced some influence on their work due to oral health problems in the past year. The most common problem was a reduced ability to concentrate at work. Generally, the participants with oral health problems tended to have work problems. The results of other variables are shown in Additional Table 5.

**Table 2:** Prevalence (%) of oral health-related work problems by oral status (*n* = 3930)

	n(%)	Presence of work problems due to oral symptoms or diseases	Specific work problems						
			Stress	Lack of focus	Lack of sleep	Lack of energy	Lack of communication due to halitosis	Lack of communication due to appearance	Lack of ability to work due to dental-related pain
Total	3930 (100.0)	6.2	5.5	5.6	5.3	5.1	4.9	4.7	5.3
Self-reported oral health									
Excellent	307 (7.8)	3.9	3.3	3.3	2.9	2.9	2.0	2.3	2.3
Very good	948 (24.1)	4.6	4.0	4.1	4.1	4.1	3.6	3.0	3.9
Good	1676 (42.6)	3.9	3.5	3.6	3.5	3.3	3.2	3.2	3.6
Fair	857 (21.8)	10.6	9.2	9.7	9.0	8.2	8.2	8.4	8.9
Poor	142 (3.6)	21.8	20.4	20.4	19.0	18.3	19.0	18.3	19.0
Number of teeth									
19 or fewer teeth	562 (14.3)	12.6	11.4	11.2	11.2	10.9	11.2	11.9	10.9
20 or more teeth	3368 (85.7)	5.1	4.5	4.7	4.4	4.1	3.8	3.5	4.3
Bleed when brush teeth									
Always	159 (4.0)	22.6	19.5	19.5	19.5	18.2	18.9	17.0	18.9
Sometimes	1607 (40.9)	7.8	6.6	7.0	6.5	6.1	6.1	5.9	6.5
Never	2164 (55.1)	3.8	3.6	3.6	3.5	3.3	2.9	3.0	3.3

Tables 3, 4 and 5 show the results of the logistic regression analysis for the association between oral health status and work problems. The odds ratios of the presence of work problems and seven work problems were high for all oral health problems. Workers with poorer oral status tended to have all specific work problems.

**Table 3:** Logistic regression analysis of the association between self-reported oral health and work problems (*n* = 3930)

		Presence of work problems due to oral symptoms or diseases		Stress		Lack of focus		Lack of sleep	
		Univariable	Multivariable	Univariable	Multivariable	Univariable	Multivariable	Univariable	Multivariable
		Odds Ratio (95% CI)	Odds Ratio (95% CI)	Odds Ratio (95% CI)	Odds Ratio (95% CI)	Odds Ratio (95% CI)	Odds Ratio (95% CI)	Odds Ratio (95% CI)	Odds Ratio (95% CI)
	Very good	1.20 (0.62;2.30)	1.14 (0.59;2.20)	1.24 (0.61;2.52)	1.21 (0.59;2.47)	1.27 (0.63;2.58)	1.23 (0.60;2.52)	1.42 (0.68;2.97)	1.38 (0.66;2.91)
Self-reported oral health	Good	1.01 (0.54;1.89)	0.92 (0.49;1.75)	1.08 (0.55;2.14)	1.03 (0.52;2.06)	1.12 (0.57;2.21)	1.07 (0.53;2.13)	1.19 (0.58;2.42)	1.13 (0.55;2.33)
(Ref: Excellent)	Fair	2.92 (1.58;5.41)*	2.13 (1.12;4.04)*	3.02 (1.54;5.90)*	2.33 (1.16;4.66)*	3.19 (1.63;6.22)*	2.46 (1.23;4.91)*	3.27 (1.62;6.60)*	2.49 (1.21;5.14)*
	Poor	6.87 (3.41;13.84)*	3.58 (1.70;7.56)*	7.62 (3.60;16.15)*	4.28 (1.93;9.51)*	7.62 (3.60;16.15)*	4.35 (1.97;9.63)*	7.77 (3.55;17.04)*	4.30 (1.87;9.86)*
		Lack of energy		Lack of communication due to halitosis		Lack of communication due to appearance		Lack of ability to work due to dental-related pain	
		Univariable	Multivariable	Univariable	Multivariable	Univariable	Multivariable	Univariable	Multivariable
		Odds Ratio (95% CI)	Odds Ratio (95% CI)	Odds Ratio (95% CI)	Odds Ratio (95% CI)	Odds Ratio (95% CI)	Odds Ratio (95% CI)	Odds Ratio (95% CI)	Odds Ratio (95% CI)
	Very good	1.42 (0.68;2.97)	1.37 (0.65;2.88)	1.87 (0.78;4.49)	1.74 (0.72;4.22)	1.30 (0.56;3.02)	1.22 (0.52;2.84)	1.74 (0.77;3.95)	1.69 (0.74;3.86)
Self-reported oral health	Good	1.12 (0.55;2.30)	1.05 (0.51;2.17)	1.67 (0.71;3.92)	1.50 (0.63;3.57)	1.40 (0.63;3.11)	1.30 (0.58;2.92)	1.59 (0.72;3.52)	1.53 (0.68;3.41)
(Ref: Excellent)	Fair	2.95 (1.45;5.97)*	2.19 (1.06;4.55)*	4.46 (1.92;10.38)*	3.09 (1.30;7.34)*	3.93 (1.79;8.64)*	2.85 (1.27;6.42)*	4.17 (1.90;9.15)*	3.21 (1.43;7.18)*
	Poor	7.42 (3.38;16.32)*	4.02 (1.75;9.28)*	11.78 (4.74;29.27)*	5.77 (2.22;14.96)*	9.61 (4.06;22.74)*	4.97 (2.00;12.32)*	10.06 (4.26;23.75)*	5.63 (2.29;13.86)*

Multivariable models adjusted for age, sex, educational attainment, income, the presence of diabetes, and industrial classifications

^*^P-value < 0.05

Even after accounting for the covariates, workers with poor oral health status tended to have a higher odds ratio for having work problems. Those with poor self-reported oral health were 3.58 (95% CI = 1.70;7.56) times more likely to have adverse work performance than those with excellent oral health after adjusting for the covariates. The odds ratio of 19 or fewer teeth compared to 20 or more teeth was 2.19 (95% CI = 1.60;3.01). The odds ratios of those who reported always and sometimes bleeding were 4.43 (95% CI = 2.75;7.12) and 1.76 (95% CI = 1.30;2.37), respectively.

## Discussion

The results of this study showed that among the participants of 3,930 workers, 6.2% had experienced some degree of interference with their work because of oral symptoms in the past year (Table 2). Even after considering covariates, the results of the logistic regression analysis showed that workers who experienced poorer self-reported oral health, had fewer remaining teeth, and had frequent gum bleeding when tooth brushing, tended to have a higher odds ratio of less efficiency or problems performing their work than those without any oral health problems (Tables 3, 4 and 5). Consequently, the results of our study suggest that oral health is associated with various types of work problems. The present results are consistent with previous studies that showed the association between poorer oral health and worsened quality of life [6, 14, 30], and between periodontal disease or tooth loss and quality of life [31, 32].

**Table 4:** Logistic regression analysis of the association between the number of teeth and work problems (*n* = 3930)

		Presence of work problems due to oral symptoms or diseases		Stress		Lack of focus		Lack of sleep	
		Univariable	Multivariable	Univariable	Multivariable	Univariable	Multivariable	Univariable	Multivariable
		Odds Ratio (95% CI)	Odds Ratio (95% CI)	Odds Ratio (95% CI)	Odds Ratio (95% CI)	Odds Ratio (95% CI)	Odds Ratio (95% CI)	Odds Ratio (95% CI)	Odds Ratio (95% CI)
Number of teeth	19 or fewer teeth	2.67 (1.99;3.58)*	2.19 (1.60;3.01)*	2.74 (2.01;3.72)*	2.23 (1.60;3.11)*	2.55 (1.88;3.46)*	2.08 (1.49;2.90)*	2.77 (2.03;3.77)*	2.26 (1.62;3.17)*
(Ref: 20 or more teeth)									
		Lack of energy		Lack of communication due to halitosis		Lack of communication due to appearance		Lack of ability to work due to dental-related pain	
		Univariable	Multivariable	Univariable	Multivariable	Univariable	Multivariable	Univariable	Multivariable
		Odds Ratio (95% CI)	Odds Ratio (95% CI)	Odds Ratio (95% CI)	Odds Ratio (95% CI)	Odds Ratio (95% CI)	Odds Ratio (95% CI)	Odds Ratio (95% CI)	Odds Ratio (95% CI)
Number of teeth	19 or fewer teeth	2.85 (2.08;3.91)*	2.34 (1.66;3.29)*	3.20 (2.33;4.38)*	2.59 (1.84;3.65)*	3.70 (2.70;5.06)*	3.10 (2.20;4.35)*	2.69 (1.96;3.68)*	2.20 (1.57;3.09)*
(Ref: 20 or more teeth)									

Multivariable models adjusted for age, sex, educational attainment, income, the presence of diabetes, and industrial classifications

^*^P-value < 0.05

**Table 5:** Logistic regression analysis of the association between gum bleeding and work problems (*n* = 3930)

		Presence of work problems due to oral symptoms or diseases		Stress		Lack of focus		Lack of sleep	
		Univariable	Multivariable	Univariable	Multivariable	Univariable	Multivariable	Univariable	Multivariable
		Odds Ratio (95% CI)	Odds Ratio (95% CI)	Odds Ratio (95% CI)	Odds Ratio (95% CI)	Odds Ratio (95% CI)	Odds Ratio (95% CI)	Odds Ratio (95% CI)	Odds Ratio (95% CI)
Bleed when brushing teeth	Always	7.34 (4.77;11.30)*	4.43 (2.75;7.12)*	6.48 (4.12;10.19)*	3.71 (2.24;6.12)*	6.48 (4.12;10.19)*	3.76 (2.28;6.19)*	6.75 (4.28;10.63)*	3.89 (2.36;6.44)*
(Ref: Never)	Sometimes	2.12 (1.59;2.81)*	1.76 (1.30;2.37)*	1.89 (1.40;2.55)*	1.54 (1.13;2.11)*	2.02 (1.50;2.72)*	1.65 (1.21;2.25)*	1.93 (1.42;2.61)*	1.58 (1.15;2.17)*
		Lack of energy		Lack of communication due to halitosis		Lack of communication due to appearance		Lack of ability to work due to dental-related pain	
		Univariable	Multivariable	Univariable	Multivariable	Univariable	Multivariable	Univariable	Multivariable
		Odds Ratio (95% CI)	Odds Ratio (95% CI)	Odds Ratio (95% CI)	Odds Ratio (95% CI)	Odds Ratio (95% CI)	Odds Ratio (95% CI)	Odds Ratio (95% CI)	Odds Ratio (95% CI)
Bleed when brushing teeth	Always	6.48 (4.07;10.33)*	3.96 (2.37;6.63)*	7.76 (4.85;12.41)*	4.55 (2.71;7.66)*	6.71 (4.14;10.88)*	3.71 (2.17;6.35)*	6.76 (4.26;10.72)*	3.91 (2.35;6.50)*
(Ref: Never)	Sometimes	1.89 (1.38;2.58)*	1.59 (1.15;2.21)*	2.17 (1.57;2.99)*	1.78 (1.27;2.50)*	2.06 (1.49;2.85)*	1.63 (1.16;2.29)*	2.03 (1.49;2.76)*	1.65 (1.20;2.27)*

Multivariable models adjusted for age, sex, educational attainment, income, the presence of diabetes, and industrial classifications

^*^P-value < 0.05

Regarding the impact on work performance, a previous study evaluating absenteeism and presenteeism due to oral health problems reported a negative association between oral health problems and work performance [16]. However, significant presenteeism was reported due to the presence of periodontal diseases (over 4 mm deep periodontal pockets) with an odds ratio of 2.011 [16]. In this study, we added the detailed information about presenteeism related to oral health problems, including its impact on both physical and mental health as it relates to work performance. To the best of our knowledge, previous studies have not investigated detailed types of work problems caused by oral health problems. In particular, frequent gum bleeding (always bleeding) when brushing teeth showed a higher odds ratio for both physical and mental health problems related to work performance, ranging between 6.48 and 7.76 in the univariable analysis and between 3.71 and 4.55 in the multivariable analysis (Table 5).

Moreover, our study results suggest the possibility that general oral health status also relates to both physical and mental health problems regarding work presenteeism. The odds ratio calculated by logistic regression analysis showed higher values for “poor” self-reported oral health than for “very good”, “good”, or “fair” self-reported oral health (Table 3). The odds ratios of work problems for “very good” self-reported oral health ranged between 1.20 and 1.87 in the univariable analysis and between 1.14 and 1.74 in the multivariable analysis, and they were not significant. Nevertheless, the odds ratios for “poor” self-reported oral health ranged between 6.87 and 11.78 in the univariable analysis, and between 3.58 and 5.77 in the multivariable analysis. These results highlight the significance of the analysis for both physical and mental health problems for work presenteeism, which were not reported in previous studies.

There are several possible mechanisms for the impact of oral symptoms and diseases on work presenteeism. First, oral health affects social relationships. Poor dental appearance due to tooth loss decreases social interactions [4]. Halitosis, often related to poor oral hygiene, also decreases in-person communication with others [3, 15, 33]. Our results showed significant association between oral health status and concerns about one’s appearance, as well as “bad breath”, resulting in work problems. Communication problems due to poor oral health also contribute to the deterioration of work performance. Second, acute dental pain can have a significant impact on work presenteeism. Previous studies have reported that dental pain causes stress and decreased QOL [1, 2, 13]. Our study also found that dental pain leads to presenteeism (Tables 3, 4 and 5). Stress and decreased quality of life due to dental pain are thought to adversely affect work performance. Third, the disruption of sleep due to oral health problems affects work performance. Our questionnaire asked about sleep problems due to oral health, and compared individuals with poor oral health to those with excellent oral health. Those with poor oral health were prone to sleep problems and were more likely to have work problems. In previous studies, it has been reported that orofacial pain causes sleep disorders [34, 35]. Sleep problems are known to decrease work performance and to increase presenteeism [36, 37].

The results of the logistic regression analysis clearly showed that poor oral health status was significantly associated with various types of work problems and increased the odds ratios of these problems. This study has some limitations. We used a self-report questionnaire, did not assess oral status clinically and did not validate the questionnaire. In a self-administered questionnaire, inaccurate responses are likely to occur, which can lead to misclassifications. This may result in a bias toward the overestimation or underestimation of the association between oral health status and work problems, or widening the confidence interval of the association. It is not possible to determine the direction of the bias. However, the present finding obtained from logistic regression analysis that the more oral health problems, the more work problems related to oral health problems appears theoretically valid.

This study was a cross-sectional study, and the temporal relationship could not be observed. While we believe in the possibility of reverse causality that oral health related work problems cause oral health problems, this is not a realistic supposition. The strength of this study is that the observed associations were strong. Some odds ratios exceeded 3 to 5, making it less likely that the observed associations could be explained by unmeasured confounders.

## Conclusion

The present study suggested that oral health problems are relevant to various types of work problems. Since these multiple work problems due to oral status could lead to productivity loss, the improvement of oral health in workers through the oral health promotion policies and oral health education should be implemented in work environments.

## Supplementary Information

**Additional file 1: Additional Table 1.** The distribution of the 5-point scale of specific oral health-related work problems (*n*=3930). **Additional Table 2.** Sensitivity analysis of the association between self-reported oral health and work problems (*n*=3930). **Additional Table 3.** Sensitivity analysis of the association between the number of teeth and work problems (*n*=3930). **Additional Table 4.** Sensitivity analysis of the association between the gum bleeding and work problems (*n*=3930). **Additional Table 5**. Prevalence (%) of oral health-related work problems by oral status (*n*=3930).

## Data Availability

The datasets analyzed during the current study are not publicly available due to privacy or ethical restrictions but are available from the corresponding author on reasonable request.
