# Supplementary material for: Association of oral health with various work problems: a cross-sectional study of Japanese workers
Source: BMC Oral Health. 2023 Jul 15;23:488. doi: 10.1186/s12903-023-03196-4 (PMC10349489; doi:10.1186/s12903-023-03196-4)
Supplement: Supplementary file 1 — Additional file 1: Additional Table 1. The distribution of the 5-point scale of specific oral health-related work problems (n=3930). Additional Table 2. Sensitivity analysis of the association between self-reported oral health and work problems (n=3930). Additional Table 3. Sensitivity analysis of the association between the number of teeth and work problems (n=3930). Additional Table 4. Sensitivity analysis of the association between the gum bleeding and work problems (n=3930). Additional Table 5. Prevalence (%) of oral health-related work problems by oral status (n=3930). [file 12903_2023_3196_MOESM1_ESM.docx]

| 5-point scale* | Stress | Lack of focus | Lack of sleep | Lack of energy | Lack of communication due to halitosis | Lack of communication due to appearance | Lack of ability to work due to dental-related pain |
| --- | --- | --- | --- | --- | --- | --- | --- |
|  | n (%) | n (%) | n (%) | n (%) | n (%) | n (%) | n (%) |
| 1 | 3715 (94.5) | 3708 (94.4) | 3720 (94.7) | 3731 (94.9) | 3739 (95.1) | 3744 (95.3) | 3723 (94.7) |
| 2 | 30 (0.8) | 18 (0.5) | 51 (1.3) | 60 (1.5) | 72 (1.8) | 65 (1.7) | 49 (1.3) |
| 3 | 71 (1.8) | 82 (2.1) | 72 (1.8) | 68 (1.7) | 60 (1.5) | 54 (1.4) | 57 (1.5) |
| 4 | 57 (1.5) | 70 (1.8) | 47 (1.2) | 39 (1.0) | 38 (1.0) | 42 (1.1) | 55 (1.4) |
| 5 | 57 (1.5) | 52 (1.3) | 40 (1.0) | 32 (0.8) | 21 (0.5) | 25 (0.6) | 46 (1.2) |

Additional Table 1. The distribution of the 5-point scale of specific oral health-related work problems (*n*=3930)

*: From 1: a great impact to 5: no impact

Additional Table 2. Sensitivity analysis of the association between self-reported oral health and work problems (*n*=3930)

|  |  | Stress | | Lack of focus | | Lack of sleep | | Lack of energy | |
| --- | --- | --- | --- | --- | --- | --- | --- | --- | --- |
|  | | Univariable | Multivariable | Univariable | Multivariable | Univariable | Multivariable | Univariable | Multivariable |
|  | | Odds Ratio (95% CI) | Odds Ratio (95% CI) | Odds Ratio (95% CI) | Odds Ratio (95% CI) | Odds Ratio (95% CI) | Odds Ratio (95% CI) | Odds Ratio (95% CI) | Odds Ratio (95% CI) |
|  | Very good | 1.25 (0.61 ;2.53) | 1.21 (0.59 ;2.48) | 1.28 (0.63 ;2.60) | 1.25 (0.61 ;2.56) | 1.42 (0.68 ;2.96) | 1.40 (0.66 ;2.94) | 1.42 (0.68 ;2.97) | 1.39 (0.66 ;2.92) |
| Self-reported oral health | Good | 1.07 (0.54 ;2.12) | 1.02 (0.51 ;2.04) | 1.11 (0.56 ;2.19) | 1.06 (0.53 ;2.12) | 1.17 (0.58 ;2.39) | 1.12 (0.54 ;2.31) | 1.12 (0.55 ;2.28) | 1.05 (0.51 ;2.17) |
| (Ref:　Excellent) | Fair | 2.97 (1.52 ;5.80)* | 2.29 (1.15 ;4.58)* | 3.14 (1.61 ;6.14)* | 2.44 (1.22 ;4.86)* | 3.24 (1.60 ;6.55)* | 2.47 (1.19 ;5.09)* | 2.92 (1.44 ;5.92)* | 2.18 (1.05 ;4.52)* |
|  | Poor | 7.98 (3.77 ;16.90)* | 4.51 (2.03 ;10.02)* | 8.02 (3.79 ;16.99)* | 4.70 (2.12 ;10.40)* | 7.85 (3.59 ;17.19)* | 4.29 (1.87 ;9.84)* | 7.66 (3.48 ;16.82)* | 4.15 (1.80 ;9.54)* |
|  |  | Lack of communication due to halitosis | | Lack of communication due to appearance | | Lack of ability to work due to dental-related pain | |  |  |
|  | | Univariable | Multivariable | Univariable | Multivariable | Univariable | Multivariable |  |  |
|  | | Odds Ratio (95% CI) | Odds Ratio (95% CI) | Odds Ratio (95% CI) | Odds Ratio (95% CI) | Odds Ratio (95% CI) | Odds Ratio (95% CI) |  |  |
|  | Very good | 1.86 (0.78 ;4.48) | 1.75 (0.72 ;4.24) | 1.30 (0.56 ;3.01) | 1.22 (0.52 ;2.83) | 1.74 (0.77 ;3.94) | 1.70 (0.75 ;3.88) |  |  |
| Self-reported oral health | Good | 1.67 (0.71 ;3.91) | 1.52 (0.64 ;3.61) | 1.39 (0.63 ;3.09) | 1.30 (0.58 ;2.92) | 1.57 (0.71 ;3.47) | 1.51 (0.68 ;3.38) |  |  |
| (Ref: Excellent) | Fair | 4.44 (1.91 ;10.33)* | 3.09 (1.30 ;7.33)* | 3.92 (1.79 ;8.62)* | 2.86 (1.27 ;6.41)* | 4.16 (1.90 ;9.12)* | 3.22 (1.44 ;7.20)* |  |  |
|  | Poor | 12.56 (5.05 ;31.20)* | 6.15 (2.37 ;15.96)* | 10.08 (4.26 ;23.86)* | 5.34 (2.16 ;13.19)* | 10.44 (4.43 ;24.63)* | 5.95 (2.42 ;14.62)* |  |  |

Multivariable models adjusted for age, sex, educational attainment, income, the presence of diabetes, and industrial classifications.

*: P-value < 0.05

Additional Table 3. Sensitivity analysis of the association between the number of teeth and work problems (*n*=3930)

|  |  | Stress | | Lack of focus | | Lack of sleep | | Lack of energy | |
| --- | --- | --- | --- | --- | --- | --- | --- | --- | --- |
|  | | Univariable | Multivariable | Univariable | Multivariable | Univariable | Multivariable | Univariable | Multivariable |
|  | | Odds Ratio (95% CI) | Odds Ratio (95% CI) | Odds Ratio (95% CI) | Odds Ratio (95% CI) | Odds Ratio (95% CI) | Odds Ratio (95% CI) | Odds Ratio (95% CI) | Odds Ratio (95% CI) |
| Number of teeth | 19 or fewer teeth | 2.73 (2.01 ;3.70)* | 2.20 (1.58 ;3.06)* | 2.50 (1.84 ;3.39)* | 2.00 (1.44 ;2.78)* | 2.75 (2.02 ;3.75)* | 2.23 (1.60 ;3.11)* | 2.85 (2.08 ;3.90)* | 2.32 (1.65 ;3.26)* |
| (Ref: 20 or more teeth) |  |  |  |  |  |  |  |  |  |
|  |  | Lack of communication due to halitosis | | Lack of communication due to appearance | | Lack of ability to work due to dental-related pain | |  |  |
|  | | Univariable | Multivariable | Univariable | Multivariable | Univariable | Multivariable |  |  |
|  | | Odds Ratio (95% CI) | Odds Ratio (95% CI) | Odds Ratio (95% CI) | Odds Ratio (95% CI) | Odds Ratio (95% CI) | Odds Ratio (95% CI) |  |  |
| Number of teeth | 19 or fewer teeth | 3.23 (2.36 ;4.43)* | 2.60 (1.85 ;3.66)* | 3.76 (2.75 ;5.15)* | 3.21 (2.29 ;4.51)* | 2.66 (1.95 ;3.64)* | 2.15 (1.53 ;3.01)* |  |  |
| (Ref: 20 or more teeth) |  |  |  |  |  |  |  |  |  |

Multivariable models adjusted for age, sex, educational attainment, income, the presence of diabetes, and industrial classifications.

*: P-value < 0.05

Additional Table 4. Sensitivity analysis of the association between the gum bleeding and work problems (*n*=3930)

|  |  | Stress | | Lack of focus | | Lack of sleep | | Lack of energy | |
| --- | --- | --- | --- | --- | --- | --- | --- | --- | --- |
|  | | Univariable | Multivariable | Univariable | Multivariable | Univariable | Multivariable | Univariable | Multivariable |
|  | | Odds Ratio (95% CI) | Odds Ratio (95% CI) | Odds Ratio (95% CI) | Odds Ratio (95% CI) | Odds Ratio (95% CI) | Odds Ratio (95% CI) | Odds Ratio (95% CI) | Odds Ratio (95% CI) |
| Bleed when brushing teeth | Always | 6.45 (4.11 ;10.12)* | 3.67 (2.23 ;6.05)* | 6.56 (4.18 ;10.29)* | 3.82 (2.32 ;6.27)* | 6.94 (4.41 ;10.93)* | 4.02 (2.44 ;6.64)* | 6.57 (4.13 ;10.46)* | 4.10 (2.46 ;6.84)* |
| (Ref: Never) | Sometimes | 1.89 (1.40 ;2.55)* | 1.55 (1.13 ;2.12)* | 2.03 (1.51 ;2.72)* | 1.66 (1.22 ;2.27)* | 1.94 (1.43 ;2.62)* | 1.58 (1.15 ;2.18)* | 1.90 (1.39 ;2.59)* | 1.61 (1.17 ;2.23)* |
|  |  | Lack of communication due to halitosis | | Lack of communication due to appearance | | Lack of ability to work due to dental-related pain | |  |  |
|  | | Univariable | Multivariable | Univariable | Multivariable | Univariable | Multivariable |  |  |
|  | | Odds Ratio (95% CI) | Odds Ratio (95% CI) | Odds Ratio (95% CI) | Odds Ratio (95% CI) | Odds Ratio (95% CI) | Odds Ratio (95% CI) |  |  |
| Bleed when brushing teeth | Always | 8.21 (5.13 ;13.12)* | 4.81 (2.87 ;8.09)* | 6.83 (4.22 ;11.06)* | 3.77 (2.21 ;6.44)* | 6.80 (4.29 ;10.77)* | 3.96 (2.39 ;6.56)* |  |  |
| (Ref: Never) | Sometimes | 2.17 (1.57 ;2.99)* | 1.79 (1.27 ;2.50)* | 2.06 (1.49 ;2.85)* | 1.63 (1.16 ;2.28)* | 2.04 (1.50 ;2.77)* | 1.66 (1.20 ;2.29)* |  |  |

Multivariable models adjusted for age, sex, educational attainment, income, the presence of diabetes, and industrial classifications.

*: P-value < 0.05

Additional Table 5. Prevalence (%) of oral health-related work problems by oral status (*n*=3930)

|  |  | n(%) | Presence of work problems due to oral symptoms or diseases | Specific work problems | |  |  |  |  |  |
| --- | --- | --- | --- | --- | --- | --- | --- | --- | --- | --- |
|  |  |  |  | Stress | Lack of focus | Lack of sleep | Lack　of energy | Lack of communication due to halitosis | Lack of communication due to appearance | Lack of ability to work due to dental-related pain |
| Total | | 3930 (100.0) | 6.2 | 5.5 | 5.6 | 5.3 | 5.1 | 4.9 | 4.7 | 5.3 |
| Self-reported oral health | Excellent | 307 (7.8) | 3.9 | 3.3 | 3.3 | 2.9 | 2.9 | 2.0 | 2.3 | 2.3 |
|  | Very good | 948 (24.1) | 4.6 | 4.0 | 4.1 | 4.1 | 4.1 | 3.6 | 3.0 | 3.9 |
|  | Good | 1676 (42.6) | 3.9 | 3.5 | 3.6 | 3.5 | 3.3 | 3.2 | 3.2 | 3.6 |
|  | Fair | 857 (21.8) | 10.6 | 9.2 | 9.7 | 9.0 | 8.2 | 8.2 | 8.4 | 8.9 |
|  | Poor | 142 (3.6) | 21.8 | 20.4 | 20.4 | 19.0 | 18.3 | 19.0 | 18.3 | 19.0 |
| Number of teeth | 19 or fewer teeth | 562 (14.3) | 12.6 | 11.4 | 11.2 | 11.2 | 10.9 | 11.2 | 11.9 | 10.9 |
|  | 20 or more teeth | 3368 (85.7) | 5.1 | 4.5 | 4.7 | 4.4 | 4.1 | 3.8 | 3.5 | 4.3 |
| Bleed when brush teeth | Always | 159 (4.0) | 22.6 | 19.5 | 19.5 | 19.5 | 18.2 | 18.9 | 17.0 | 18.9 |
|  | Sometimes | 1607 (40.9) | 7.8 | 6.6 | 7.0 | 6.5 | 6.1 | 6.1 | 5.9 | 6.5 |
|  | Never | 2164 (55.1) | 3.8 | 3.6 | 3.6 | 3.5 | 3.3 | 2.9 | 3.0 | 3.3 |
| Age | Under 30 | 625 (15.9) | 5.9 | 5.3 | 5.4 | 5.4 | 4.8 | 4.3 | 4.2 | 5.0 |
|  | 30〜39 | 969 (24.7) | 6.5 | 5.7 | 5.9 | 5.7 | 5.4 | 4.9 | 5.1 | 5.8 |
|  | 40〜49 | 1011 (25.7) | 6.7 | 5.9 | 6.0 | 5.6 | 5.1 | 5.6 | 5.2 | 5.5 |
|  | 50〜59 | 888 (22.6) | 6.4 | 5.6 | 6.0 | 5.2 | 5.3 | 4.7 | 4.7 | 5.3 |
|  | Over 60 | 437 (11.1) | 4.3 | 3.9 | 3.9 | 4.1 | 4.1 | 4.1 | 3.7 | 3.9 |
| Sex | Male | 2057 (52.3) | 5.8 | 5.2 | 5.3 | 5.2 | 4.8 | 4.7 | 4.6 | 5.1 |
|  | female | 1873 (47.7) | 6.7 | 5.8 | 6.0 | 5.5 | 5.3 | 5.0 | 4.9 | 5.5 |
| Educational attainment | 1* | 1416 (36.0) | 7.6 | 6.8 | 6.9 | 6.5 | 5.8 | 5.6 | 5.4 | 6.5 |
|  | 2* | 839 (21.3) | 6.3 | 5.4 | 5.5 | 5.4 | 5.5 | 4.8 | 4.9 | 5.1 |
|  | 3* | 1675 (42.6) | 5.0 | 4.4 | 4.7 | 4.4 | 4.2 | 4.3 | 4.1 | 4.3 |
| Income | 2< million yen | 577 (14.7) | 5.7 | 4.7 | 4.9 | 5.0 | 4.7 | 5.0 | 4.3 | 4.5 |
|  | 2≥to <4 million yen | 1479 (37.6) | 5.8 | 5.3 | 5.3 | 5.1 | 5.1 | 4.7 | 4.7 | 4.9 |
|  | 4≥to<6 million yen | 810 (20.6) | 8.9 | 7.7 | 7.9 | 7.4 | 6.7 | 6.7 | 6.8 | 7.7 |
|  | 6≥to<8 million yen | 299 (7.6) | 5.4 | 4.7 | 5.4 | 4.7 | 4.3 | 4.0 | 3.7 | 5.0 |
|  | 8≥million yen | 210 (5.3) | 2.9 | 2.9 | 2.9 | 2.9 | 2.9 | 2.9 | 2.4 | 2.9 |
|  | Unknown | 555 (14.1) | 5.6 | 4.9 | 5.2 | 4.5 | 4.1 | 3.8 | 3.8 | 4.7 |
| Diabetes | Absence | 3806 (96.8) | 6.1 | 5.4 | 5.6 | 5.2 | 4.9 | 4.7 | 4.6 | 5.2 |
|  | Presence | 124 (3.2) | 9.7 | 8.1 | 8.1 | 8.9 | 8.9 | 9.7 | 8.1 | 8.1 |
| Industry | Primary industry | 305 (7.8) | 7.2 | 6.9 | 6.9 | 6.9 | 6.6 | 5.2 | 4.9 | 6.6 |
|  | Secondary industry | 1097 (27.9) | 6.0 | 5.1 | 5.1 | 5.0 | 4.5 | 4.3 | 4.2 | 4.9 |
|  | Tertiary industry | 2528 (64.3) | 6.2 | 5.5 | 5.7 | 5.3 | 5.1 | 5.1 | 4.9 | 5.3 |

1*: Elementary and secondary school graduate

2*: Vocational school or junior college graduate

3*: University graduate, master's degree or doctoral degree, other
